# Placental mesenchymal stem cells ameliorate NLRP3 inflammasome-induced ovarian insufficiency by modulating macrophage M2 polarization

**DOI:** 10.1186/s13048-023-01136-y

**Published:** 2023-03-21

**Authors:** Dongmei Chen, Na Hu, Shasha Xing, Li Yang, Feiyan Zhang, Songlin Guo, Shudan Liu, Xiaona Ma, Xueyun Liang, Huiming Ma

**Affiliations:** 1 Key Laboratory of Stem Cell and Regenerative Medicine, Institute of Medical Sciences, General Hospital of Ningxia Medical University, Yinchuan, Ningxia 750004 China; 2 Key Laboratory of Fertility Preservation and Maintenance of Ministry of Education, Ningxia Medical University, Yinchuan, 750004 China; 3 Laboratory Animal Center, Ningxia Medical University, Yinchuan, 750004 China

**Keywords:** Placental mesenchymal stem cells, Premature ovarian insufficiency, Macrophage, Inflammasome, NLRP3, Interferon-γ, Pyroptosis

## Abstract

**Background:**

Premature ovarian insufficiency (POI) is a common clinical problem, however, there are currently no effective therapies. Pyroptosis induced by the NLRP3 inflammasome is considered a possible mechanism of POI. Placental mesenchymal stem cells (PMSCs) have excellent immunomodulatory potential and offer a promising method for treating POI.

**Methods:**

Female Sprague–Dawley rats were randomly divided into four treatment groups: control (no POI), POI with no PMSCs, POI with PMSCs transplant, and POI with hormones (estrogen + progesterone) as positive control. POI was induced by exposure to 4-vinylcyclohexene diepoxide (VCD) for 15 days. After four weeks, all animals were euthanized and examined for pathology. Hormone levels were measured and ovarian function was evaluated in relation to the estrous cycle. Levels of NLRP3 inflammasome pathway proteins were determined by immunohistochemistry and western blot.

**Results:**

VCD significantly damaged rat follicles at different estrous stages. Injection of human PMSCs improved ovarian function and reproductive ability of POI rats compared to the sham and hormone groups. Our data also showed that PMSCs markedly suppress cell pyroptosis via downregulation of the NLRP3 inflammasome, caspase-1, IL-1β and IL-18 compared to the other two groups. The human PMSCs increased the expression of IL-4 and IL-10 and decreased pro-inflammatory factors by phenotypic changes in macrophages.

**Conclusions:**

Our findings revealed a novel mechanism of follicular dysfunction and ovarian fibrosis via activation of the NLRP3 inflammasome followed by secretion of pro-inflammatory factors. Transplantation of PMSCs into POI rats suppressed pro-inflammatory factor production, NLRP3 inflammasome formation and pyroptosis, and improved ovarian function.

## Background

The low estrogen levels and loss of female fertility caused by premature ovarian insufficiency (POI) can lead to serious disease. The pathogenesis of POI is currently unclear, and there is no effective method to restore ovarian function. Women with POI develop amenorrhea before the age of 40, accompanied by endocrine symptoms such as increased Follicle stimulating hormone (FSH) levels (FSH ≥ 25 U/L) and decreased estrogen levels [1]. Clinical manifestations vary in severity, but include night sweats, poor sleep, mood changes, inattention, osteoporosis, fluctuations in blood lipids and blood pressure, and cardiovascular system diseases. Hormone replacement therapy, as a first-line clinical treatment, can reduce the symptoms caused by estrogen deficiency, but there is still no method for restoring follicular development in failed ovaries [2]. In recent years, many studies have suggested that transplantation of stem cells may be able to restore ovarian function [3]. When mesenchymal stem cells (MSCs) were used to treat POI, there was a decrease in apoptosis of ovarian granulosa cells (GCs), recovery of ovarian function, and increased levels of sex hormones [4, 5]; however, the underlying mechanism remained elusive. Human placenta is a valuable biological resource and a promising source of stem cells. Recent reports suggested that human placental mesenchymal stem cells (PMSCs) can restore ovarian function by exerting anti-inflammatory and tissue regenerative effects, the mechanisms of which mainly focus on improving the ovarian microenvironment [6]. Fibrosis and functional disorders are the result of dysregulated tissue repair responses to ovarian tissue damage, especially in the process of chronic inflammation [7]. Inflammatory cell death (pyroptosis) induced by NLRP3 inflammasomes is considered a possible mechanism of POI [8]. We previously found that a CD200^+^ subset within PMSCs presented a stronger immunomodulatory potential [9]. Whether PMSCs can reduce pyroptosis in ovarian tissue induced by inflammasomes, protect ovarian tissue from inflammatory damage, and reduce the occurrence of POI remains to be determined.

Inflammasomes are cytoplasmic polyprotein complexes, which mediate the host's immune response to microbial infection and cell damage. The aggregation of inflammasomes causes proteolytic cleavage of procaspase-1 to generate activated caspase-1, which can induce a pro-inflammatory form of cell death, known as pyroptosis [10]. NLRP3 is relatively nonspecific as it responds to a large number of agonists that are unrelated in origin, chemical composition, and structural properties. The activation of inflammasomes such as NLRP1, NLRP3, and AIM2 plays a key role in the process of tissue fibrosis. The maturation and release of IL-1β and IL-18 caused by inflammasome activation can lead to excessive structural destruction and organ dysfunction [11]. Studies have shown that human MSCs increased cell viability and proliferation and alleviated tissue damage by reducing NLRP3 inflammasome formation, caspase-1 activation and IL-1β maturation [12, 13]; but whether there is such a mechanism for the alleviation of POI by MSCs improving immune microenvironment remains unclear.

## Materials and methods

### Laboratory animals

Female Sprague–Dawley (SD) rats were supplied by the Laboratory Animal Center of Ningxia Medical University and housed in a specific pathogen free (SPF) area. The rats were caged individually and kept at a temperature of 23 ± 2 °C with a 12 h/12 h light/dark cycle, and food and water provided ad libitum. The rats were acclimatized for one week before starting the experiment. All procedures were implemented in accordance with the criteria for the care and use of laboratory animals of Ningxia Medical University. The protocol was approved by the Academic Committee on the Ethics of Animal Experiments of Ningxia Medical University (Permit Number: SCXK(Ning)2015–0001).

### Premature ovarian insufficiency (POI) model establishment

SD 48 female rats (eight-weeks-old; weight 230–255 g) were subjected to daily intraperitoneal injections of 80 mg/kg of VCD (Sigma-Aldrich, Germany) soluble in castor oil (MCE, China) for 15 days. The estrus cycle, plasma hormone levels (vacutainer tube for blood collection from tail veins, *n* = 15) and ovarian histomorphology (ovariectomy, *n* = 3) were used to measure the success rate for creation of the.

### Research design and sample collection

Fourty-five POI model rats were grouped according to the designated test conditions (*n* = 15). After the rats were allowed to reach a stable level of baseline plasma hormones, the rats randomly underwent either PMSCs implantation (POI + PMSCs, *n* = 15) or hormone with estradiol only (POI + E + P) (1 and 2.5 mg/kg, respectively, *n* = 15) through oral injection every 4 to 5 days to mimic the menstrual cycle of rats [14]. The saline group served as a POI model negative control (POI + saline, *n* = 15) and the had not treatment with VCD served as a normal control (*n* = 15). In PMSCs implantation group, rats were intravenously injected with 1 × 10^7^cells/rat on the first and 7th days. All animals were continuously observed for 30 days, estrous cyclicity were tested in first 10 days and last 10 days. The weight of the rats was weighed every 7 days. At the end of the experiment, the rats were fasted overnight and anesthetized by i.p. injection of 0.4 mL/kg sodium pentobarbitone (100 mg/mL), and the ovaries and uterus were taken out after the blood was sacrificed. Ovarian index = ovarian wet weight (mg) / body weight (g) × 100%. Uterine index = uterine wet weight (mg) / body weight (g) × 100%. Plasma hormone levels (17-estradiol, progesterone, FSH, and LH), assessed using blood draws, were checked before and after treatment. Limited to 1 or 2.5 mL of whole blood were drawn by vacutainer from tail veins (before treatment) or heart (after treatment), allowed to clot at room temperature and centrifuged at 4 °C to obtain serum. The rats were euthanized with CO_2_ and bilateral ovaries were removed. One ovary was fixed with 4% paraformaldehyde for histology analysis, and the other was stored at -80 °C for protein immunoblotting.

### Estrous cycle characterization

Vaginal exfoliation cytology smears were obtained from the experimental rats. Animals were immobilized, and 0.1 ml of normal saline in a Pasteur pipet was gently inserted 5–6 mm into the vagina, aspirated 2–3 times, and examined under a microscope. The estrous cycle was categorized as follows: (1) pre-estrus with large numbers of small nucleated epithelial cells, individual or in clusters; (2) estrus with large numbers of irregular keratinocytes and small numbers of nucleated epithelial cells; (3) post-estrus with keratinocytes, nucleated epithelial cells and leukocytes in equal proportions; and (4) inter-estrus with large numbers of leukocytes.

### Isolation and culture of human placental MSCs

Human placental tissue was obtained following informed consent from healthy volunteers who tested negative for HIV-I, hepatitis B, hepatitis C, cytomegalovirus, rubella virus and herpes simplex virus. The acquisition protocol and the informed consent document were approved by the Institutional Ethics Committee of the general hospital of Ningxia medical university. Placental chorionic membranes were mechanically separated from the fetal side, cut into 1 mm^3^ pieces, and washed with phosphate buffered saline (PBS). The tissue pieces were incubated for 60 min at 37 °C with MSC-ACF tissue digestion mix (VivaCell Biosciences, Shanghai, China). The program of tissue dissociation was executed on the GentleMACS Octo-dissociator with heaters (Miltenyi Biotec, Germany). The digested tissue was washed twice with PBS, sequentially filtered through a 70 μm filter and centrifuged at 300 g for 5 min at room temperature. The pelleted cells were seeded in 75 cm^2^ culture flasks containing UltraCulture™ medium (Lonza, Grand Island, NY, USA) supplemented with Ultroser G serum substitute (Pall, USA) and 2 mM GlutaMAX™(Gibco) and incubated at 37 °C in 5% CO_2_. After 4–5 passages, the cells were tested for MSC surface markers by flow cytometry and used for transplantation in POI rats.

### Labeling of human placental MSCs with quantum dots (QDs)

On the day before cell transplantation, PMSCs were labeled with quantum dots (QDs) (Thermo fisher, USA) by direct endocytosis. Briefly, PMSCs were washed twice in PBS, trypsin digested into cell suspensions, and 100 μL of serum-free cell culture medium containing 20 nM QDs was added to cells and incubated for 1 h at 37 °C. After removal of the medium containing the QDs, the cells were washed twice in PBS and incubated in complete cell culture medium for up to 24 h. To enable observation of the intracellular distribution of QDs in cultured PMSCs, the PMSC were labeled with Hoechst 33342 (Beyotime, Shanghai, China). Fluorescence images were acquired with an Olympus imaging system (excitation 385 nm, emission 655 nm) (Olympus FV100, Olympus, Japan).

### In vivoimaging

Labeled PMSCs (1 × 10^6^ /ml) were transplanted into POI model rats via tail vein injection. Rats were euthanized and perfused with saline at three days after PMSC injection. Ovaries and uteri were quickly removed and imaged with an IVIS Lumina III in vivo imaging system (PerkinElmer, USA). Fluorescence images were captured at 5 s intervals with the excitation and emission wavelengths set at 385 nm and 655 nm, respectively. The parameters (p/s/mm2/sr) were obtained by measuring the median fluorescence intensity (MFI) using the manufacturer’s data processing software.

### Flow cytometry assay

A flow cytometry assay for MSC markers was performed on PMSCs from passage three (P3) cultures. The cells were harvested and washed, then incubated with fluor-conjugated (PE or FITC) antibodies to CD105, CD90, CD73, CD45, CD14 and CD34 (BioLegend, San Diego, USA). The flow cytometry analysis was performed on a BD FACSCalibur™.

### In vitroanalysis of differentiation capacity

PMSCs were cultured with induction medium kits (ScienCell Research Laboratories, Carlsbad, USA) for adipogenesis, chondrogenesis and osteogenesis. For adipogenic and osteogenic differentiation, PMSCs were expanded in CellBind-treated culture dishes (Corning, CellBIND Surface). The medium was replaced after 24 h. Cells were allowed to differentiate for three weeks then fixed and stained with oil red O (ScienCell Research Laboratories, Carlsbad, USA) or alizarin red solution (ScienCell Research Laboratories, Carlsbad, USA). For chondrogenic differentiation, MSCs were cultured as pellets in complete MSC chondrogenic differentiation medium (ScienCell Research Laboratories, Carlsbad, USA), with weekly medium replacement, for four weeks. At the end of the incubation, the cell aggregates were fixed in 4% paraformaldehyde for 30 min and dehydrated with 30% sucrose solution overnight at 4 °C. The spherules were frozen within embedding agent (Tissue-Tek O.C.T. compound; Sakura, Japan) and sectioned at -20 °C using a cryostat (Leica, Germany) followed by staining with an Alcian blue kit, according to the instructions and digitally imaged under a microscope (Olympus BX51, Japan).

### Cytokine secretion assay of PMSCs

PMSCs were cultured with UltraCulture™ medium, and 20 ng/mL interferon-γ (IFNγ) was added for immune stimulation in one group. Fresh medium was added at 48 h after IFNγ stimulation and the culture medium was collected 24 h later. The relative expression levels of 1000 human cytokines were measured by a combination of 25 non-overlapping RayBio® G-Series arrays (GSH-CAA-X00-SW). After the original data was normalized by the software, it was selected for analysis by moderated *t*-statistics, adjusted *p* value (the *p* value after BH method correction) or *p* value (see Annex 4 for specific screening conditions) and logFC (fold difference in expression, there’s base 2 on the log) to screen the differentially-expressed proteins. The selection conditions were logFC > log2(1.2) and the difference threshold was 1.2.

### Monocyte induction and differentiation to macrophages

Healthy human peripheral blood (20 mL) was collected in heparinized tubes and cells were separated by centrifugation on Histopaque-1077 to obtain human peripheral blood monocytes (PBMCs), which are reactive cells. CD14-positive monocytes were sorted using an immuno-magnetic bead kit. CD14^+^ cells were cultured in IMEM with 10% FBS and 50 ng/mL human M-CSF for 7 days, for subsequent co-culture experiments or IFNγ polarization experiments.

### Macrophage co-culture to identify immune characteristics of PMSCs

The PMSCs were pretreated with mitomycin C (2 μg/ml, Sigma, Germany) and seeded in 6-well plates as stimulated cells. Macrophages were added to the upper chambers of 24 mm transwell inserts in a 6-well plate, and 20 ng/ml IFNγ was added to each well according to the instructions. After co-cultivation with PMSCs for 48 h, the macrophages were removed and stained for immunofluorescence.

### Immunostaining

Macrophages were fixed for 10 min in 4% PFA (Sigma, P6148) in PBS previously warmed to 37 °C. Cells were washed twice in PBS, and permeabilized for 5 min in 0.1% TX-100 (Thermo Fisher Scientific, BP151-100) in PBS, washed twice with PBS, and nonspecific binding was blocked with 5% bovine serum albumin (BSA) in PBS for 1 h. Cells on coverslips were incubated in a humidified lightproof container with rabbit mAb to CD68 at 1:200 dilution (ABclonal), mouse mAb to CD206 at 1:10,000 dilution (Proteintech), and p65 antibody at 1:1000 dilution (D14E12, Cell Signaling Technology) overnight at 4 °C. Cells were washed three times for 5 min each in PBS and incubated with Alexa Fluor 488-labeled goat anti-rabbit IgG or Alexa Fluor 594-labeled goat anti-mouse IgG (1:2000, Jackson), or staining solution only (negative control) for 1 h at 37 °C.Where indicated in the figure, DAPI was included in the secondary antibody incubation at 0.5 µg/ ml. Paraffin section of samples were cut and heated 2 h at 65℃. The sections were dewaxed with benzene-free clear liquid (Jiuzhoubolin, China) 20 min twiceat room temperature. The subsequent steps were the same as for cell staining.

### TUNEL assay

Paraffin sections from samples were cut and heated 2 h at 65℃. The sections were dewaxed with dimethylbenzene 20 min twice at room temperature. The one-step TUNEL in situ apoptosis detection kit (Green, AF488) (Elabscience Biotechnology Co., China) was used for apoptosis detection of tissue samples as follows. Excess moisture around the sliced ​​tissue was blotted with filter paper, 100 μL of 1 × proteinase K working solution was pipetted on each sample, and allowed to react at 37 °C for 20 min. The coverslips were washed 3 × , 5 min each, with PBS to stop the reaction, then 100 μL of TdT equilibration buffer was added dropwise to each sample, and allowed to react at 37 °C for 10–30 min. Labeled working solution (50 μL) was added to each sample and the coverslips were placed inside a humid chamber in the dark for 60 min at 37 °C. The samples were immersed in PBS and rinsed 3 times for 5 min each. Wash liquid was blotted with filter paper, and the sections were covered with DAPI working solution, and incubated at room temperature for 5 min in the dark, to counterstain the nuclei blue. The coverslips were rinsed 3 × , 10 min each with PBS and mounted cell-side down on clean glass slides using a fluorescence-compatible mounting medium.

### ELISA assay

In rats with an estrous cycle, serum hormones were detected during the proestrus. Rats without estrous cycles had serum hormones at any time. Serum anti-müllerian hormone (AMH), follicle-stimulating hormone (FSH), and estradiol (E2) concentration were determined using ELISA kits according to instructions (Shanghai JiangLai Biotechnology Co., Ltd). Ninety-six-well plates coated with antibodies were incubated with serum samples (1:10 dilution, *n* = 8) at RT for 2 h. Absorbance was measured with a microplate reader, and compared to standard curves to determine hormone concentrations.

### Histological evaluation

Tissues were fixed in 4% paraformaldehyde (PFA) at room temperature (RT) for 24 h, then dehydrated, cleared and embedded in paraffin. The tissue blocks were sectioned at 5 µm using a microtome (Leica, Germany). The sections on slides were treated with 3% hydrogen peroxide for 20 min, blocked with normal goat serum for 1 h at RT and then incubated overnight at 4 °C with primary antibodies against NLRP3, NFκB, ASC(apoptosis-associated speck-like protein containing CARD), Caspase-1, TLR4, TNF-α and IL-1β, diluted 1:500–1:1000. After washing, the sections were incubated with secondary antibody, visualized using diaminobenzidine substrate, and counterstained with hematoxylin, or hematoxylin and eosin (H&E). Images were captured by TissueFAXS CHROMA and analyzed by TissueFAXS imaging software, 7.0.

### Van Gieson (VG) staining for ovarian fibers

Dewaxed ovarian tissue sections were first stained with celestin blue (nuclei) for 5 min, washed with DIW and stained with hematoxylin for 5 min. After washing well in running tap water for 5 min, the slides were flooded with Curtis stain (saturated aqueous picric acid, 1% ponceau S, glacial acetic acid at a 9:1:1 ratio) for 5 min, dehydrate rapidly in ethanol series, cleared and mounted. The elastic fibers were stained blue-black and background was stained yellow.

### Fertility test

Each male rats (8–10 weeks) was caged with two wild-type females. Vaginal plugs were checked for copulation every morning. Once a vaginal plug was identified (day 1 postcoitus), The plugged female was separated and singly caged, and the pregnancy results were recorded. If a female generated no pups until day 22 postcoitus, it was deemed as not pregnant. Each female underwent two cycles of the above fertility test.

### Western immunoblotting

Ovarian tissue samples were placed in RIPA (Radio Immunoprecipitation Assay) lysis buffer with a cocktail of protease and phosphatase inhibitors and homogenized using a frozen tissue grinder. BCA assay was used to quantify the amount of total protein in each sample. Equal amounts (20 μg) of protein were loaded into the wells of a 10% SDS-PAGE gel and run for 2 h at 150 V. The proteins were transferred from the gel to a PVDF (Polyvinylidene Fluoride) membrane for 1 h at 300 mA. The membrane was blocked for 1 h at RT with TBST + 5% nonfat dry milk, then with appropriate dilutions of primary antibody in blocking buffer overnight at 4 °C. The membrane was washed three times with TBST, then incubated with the recommended dilution of HRP-conjugated secondary antibody in blocking buffer at RT for 1 h. The membrane was washed three times with TBST (Tris Buffered Saline with Tween® 20), 5 min each, and incubated in enhanced chemiluminescence (ECL) reagent according to manufacturer’s directions. Images were acquired using darkroom development techniques for chemiluminescence with the GE-Amersham Imager 600. Intensities of protein bands were measured using Image J. Relative protein levels were normalized to expression of GAPDH.

### Statistical analysis

SPSS 23.0 (IBM, Armonk, NY, U.S.A.) was used for statistical analyses. The Shapiro–Wilk (S-W) test was used to determine normality and lognormality of the data. Data that were normally distributed were analyzed using one-way analysis of variance (ANOVA) and Tukey's post hoc test. Data are expressed as mean ± standard deviation. Non-normally distributed continuous variables are expressed as medians and were compared using a nonparametric test. The Wilcoxon-test was used to determine whether differences were statistically significant by comparing the paired samples before and after treatment, and the Mann–Whitney test was used to determine significance by comparing the unpaired samples with the control group, before or after treatment. A *p*-value < 0.05 was considered statistically significant.

## Results

### Characterization and labeling of human PMSCs

**Fig. 1:**
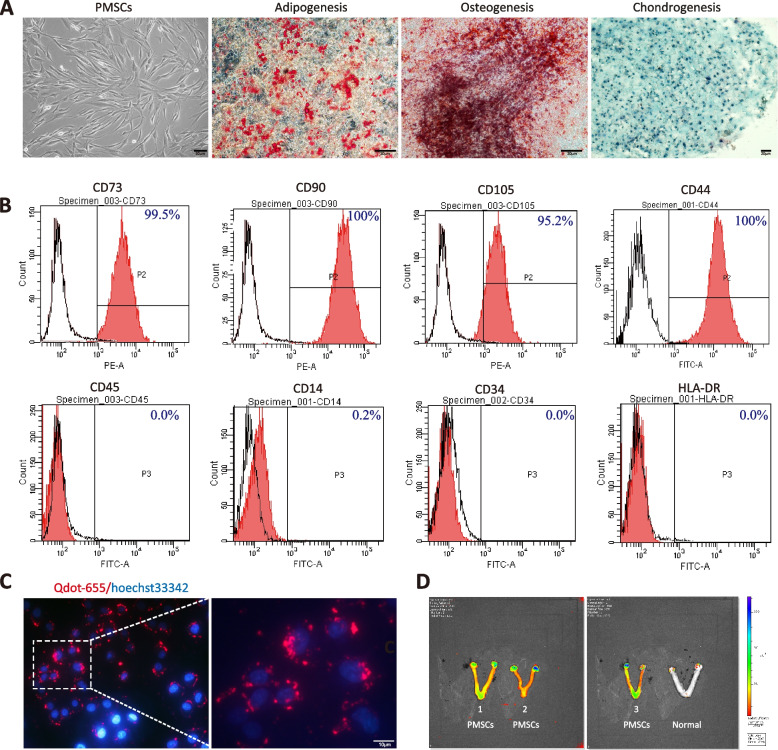
Characterization and identification of human PMSCs. **A** Morphology of PMSCs at the fourth passage. Oil Red O staining was conducted for adipogenic differentiation, alizarin red staining was used for osteogenic identification, and Alcian blue indicated chondrogenesis. Scale bar: 50 or 200 µm. **B** human PMSCs were positive for CD73, CD90, CD105, CD44, and CD90, and were negative for CD34, CD45, CD14 and HLA-DR, as shown by flow cytometry analysis. **C** Qtracker® 655-labeled PMSCs (red) counterstained with Hoechst 33342 (blue). **D** Live imaging of POI model rat ovaries and uteri transplanted with Qtracker® 655-labeled PMSCs. A separate control rat, not inoculated with cells, was imaged and is shown at the far right for comparison

Placenta-derived MSCs show typical MSC phenotype and morphology, with a characteristic spindle-like shape. Oil red O staining showed accumulated triacylglycerols indicative of adipogenesis, alizarin red S staining showed mineral deposition from osteogenesis, and Alcian blue staining showed proteoglycan from chondrogenesis in the cells (Fig. 1A). Flow cytometry analysis demonstrated that over 95% of the PMSCs retained their ability to express MSC surface immunophenotypic markers, such as CD105 (Clusters of Differentiation 105), CD73 and CD90, but lacked expression of hematopoietic markers CD34, CD14, CD45, and the MHC class II molecule, HLA-DR (Fig. 1B). Living cells labeled for 60 min with Qtracker® 655 for mesenchymal stem cell tracking (Fig. 1C). Infrared cell tracking 72 h after infusion revealed that the colonization of the labeled cells was detected in ovaries and uterus tissue (Fig. 1D). The integrated intensity of the control ovaries was 1.28 E + 07, whereas that of the MSC-transplanted ovaries was 4.16E + 07 (*p* = 0.0002, *t* test). Thus, the SEM for these differences was 2.87 E + 07 ± 3.54 E + 06 and the 95% confidence interval for the average is 2.01 E + 07 to 3.74 E + 07.

### PMSCs preserve ovarian function in VCD-induced rat POI model

**Fig. 2:**
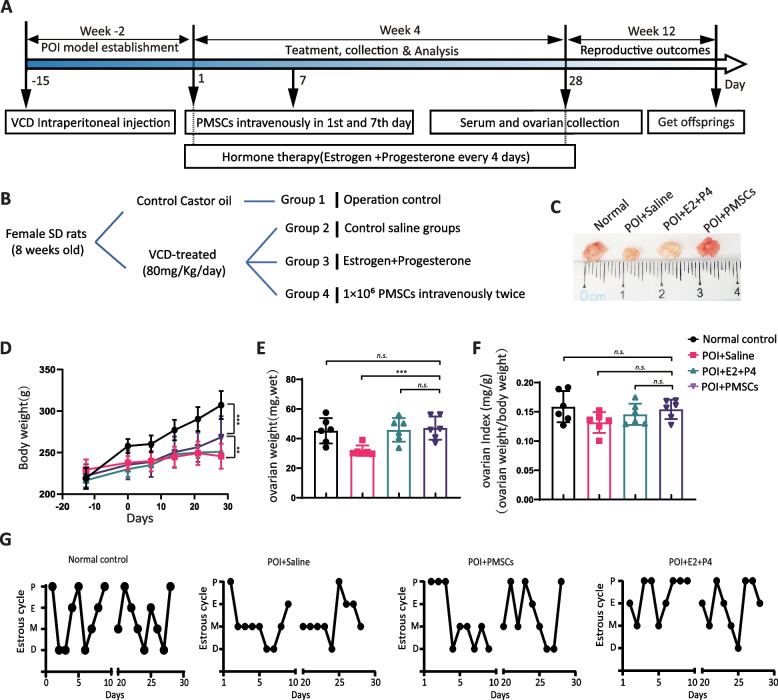
Experimental timeline and changes in ovarian morphology and function in SD rats. **A** Experimental timeline. After the POI model was established by VCD injection, seven days after the first treatment, MSC transplantation was performed by two injections of PMSCs. Estrogen and progesterone treatment was used as a positive control. **B** experimental grouping. **C** Ovarian size. **D** Body weight change. **E** Ovarian weight. **F** Ovarian index. **G** Estrous cycle was measured by exfoliated vaginal cell staining

PMSC transplantation was done after establishment of the POI model and the effects of PMSCs on ovarian function were determined. The experimental design and progression of steps are shown in Fig. 2A. Exposure to VCD led to a smaller increase in body weight and ovarian volume over time compared to control animals (Fig. 2C-D). Body and ovarian weights were restored by PMSC transplantation; the values of ovarian weight were not significantly affected by PMSCs, however (Fig. 2D-F). The estrous cycle showed a regular progression from pre-estrus, to estrus, then post-estrus and inter-estrus in 5–6 days. After the last injection of VCD, the rats’ estrous cycles were disrupted, and this resulted in a longer period of estrus. At ten days after the first injection of PMSCs, we observed a partial restoration of the estrous cycle in the MSCs group and hormone therapy group compared to the saline control group (Fig. 2G).

**Fig. 3:**
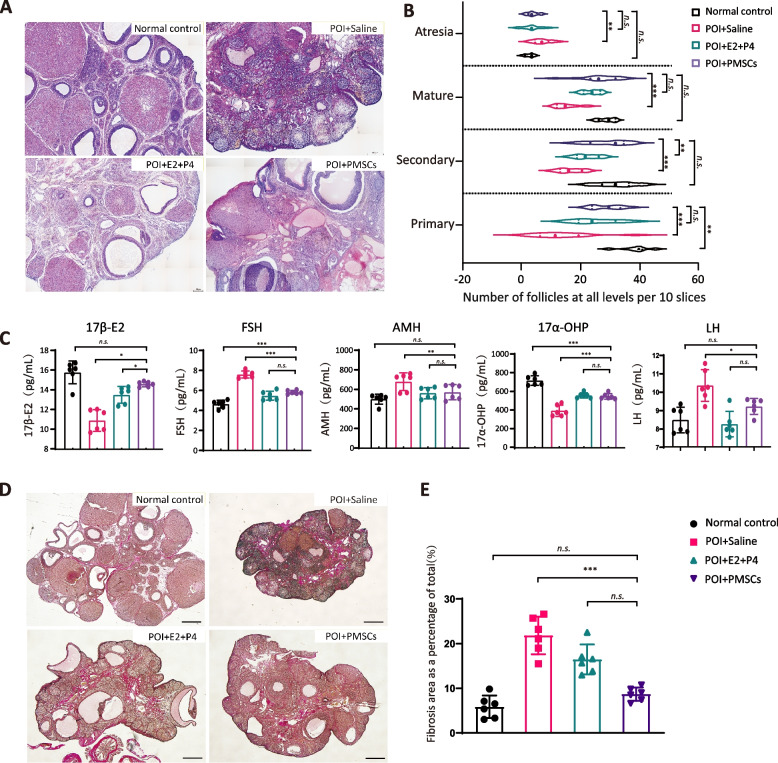
Therapeutic effects of PMSCs in a rat model of POI. **A** H&E staining of ovaries in the control and POI groups. Scale bar: 50 µm. **B** The number of follicles at different stages in each group. **C** AMH, E2, LH and FSH levels were significantly elevated in four groups. **D** The VG staining in each group. Scale bar: 200 μm. **E** Fibrosis levels were significantly elevated in four groups compared. Data are presented as the mean ± SD. *p < 0.05, **p < 0.01, ***p < 0.001, *ns*, not significant. Data are means of three independent experiments in each group. Data are presented as the mean ± SD. *p < 0.05, **p < 0.01, ***p < 0.001, *ns*, not significant. Data are means of three independent experiments in each group

At 28 days after the last injection, H&E staining showed good follicle formation at all stages in the control group, and mature follicles were overdeveloped in the hormone therapy group. The ovarian tissue structure in the POI model group was disordered, the number of early follicles in the ovaries of the rats, including primordial follicles and primary follicles were significantly decreased. There were multiple atresia follicles, and the GC layer significantly decreased (Fig. 3A). Compared to POI model group, H&E staining of ovarian sections revealed that the numbers of follicles in all stages were significantly increased in the PMSC group. The overall ovarian tissue morphology was improved along with numbers of primary and secondary follicles, while the number of atresia follicles had decreased (*p* < 0.01) (Fig. 3B). Ovarian function was evaluated in terms of follicle-stimulating hormone (FSH), estrogen (17β-E_2_), luteinizing hormone (LH), anti-Müllerian hormone (AMH) and 17-hydroxyprogesterone (17-OHP). In the POI model rats, loss of ovarian function resulted in abnormally low levels of estrogen, and AMH, but high levels of FSH. There were significant increases in serum levels of sex hormones E2 (*p* < 0.01) and AMH (*p* < 0.01) after injection of PMSCs, and FSH was effectively reduced to close to normal levels (Fig. 3C). Compared with the control group, Van Gieson staining of ovarian sections revealed that interstitial fibrosis was more severe in the POI group (Fig. 3D, E). The control group ovarian tissue showed significantly greater numbers of red and yellow collagen fibers, and the degree of fibrosis was higher than that of the normal group and PMSCs transplanted group (*p* < 0.01). The difference between control and the hormone group was not statistically significant (*p* > 0.05).

**Fig. 4:**
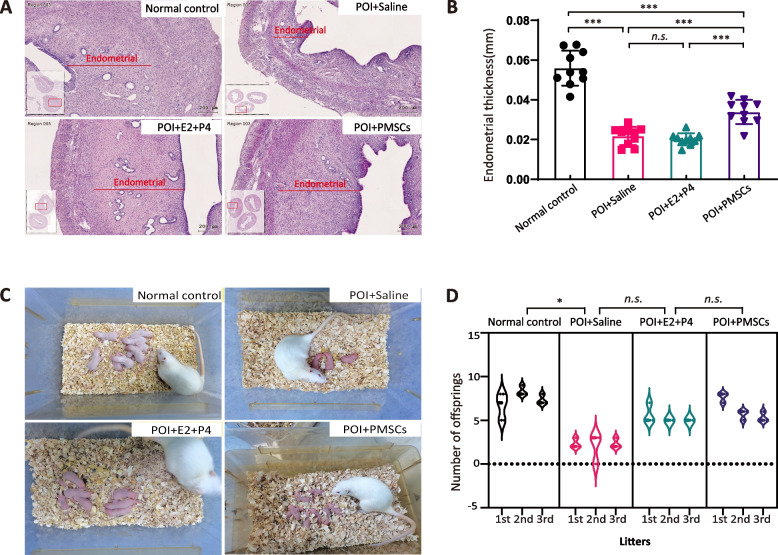
Endometrial regeneration and production of offspring by PMSC treatment. **A** H&E staining (scale bar, 200 μm) with **B** endometrial thickness of uteri. **C** The morphological images and **D** quantitative analysis of offspring. Data are presented as the mean ± SD. *p < 0.05, **p < 0.01, ***p < 0.001, *ns*, not significant. Data are means of three independent experiments in each group

The PMSC-implanted group and the hormone group exhibited regenerated endometrium similar to the levels of the normal group compared to the saline group (Fig. 4A, B). There were more glands in the uterus, and the myometrium was thicker in the normal, the PMSC-implanted, and the hormone group (Fig. 4A). Fertility results of the four groups showed that PMSC transplantation significantly improved the reproductive functions of POI rats (Fig. 4C). The number of offspring was significantly suppressed in the POI group compared with the normal group (*p* < 0.001). In contrast, treatment with PMSCs significantly increased the number of offspring compared with the POI group without stem cells (*p* < 0.05) (Fig. 4D). However, after four weeks of hormone therapy, the first litter was similar to those of the normal group, but the number of offspring decreased in the latter two litters. The time-to-birth in the POI group was significantly prolonged, but this effect was reversed by PMSCs transplantation. Taken together, these findings showed that administering PMSCs can greatly improve ovarian function and reproductive ability in POI rats.

### PMSCs alleviate pro-inflammatory cytokine secretion as well as enhances M2 macrophage differentiation

**Fig. 5:**
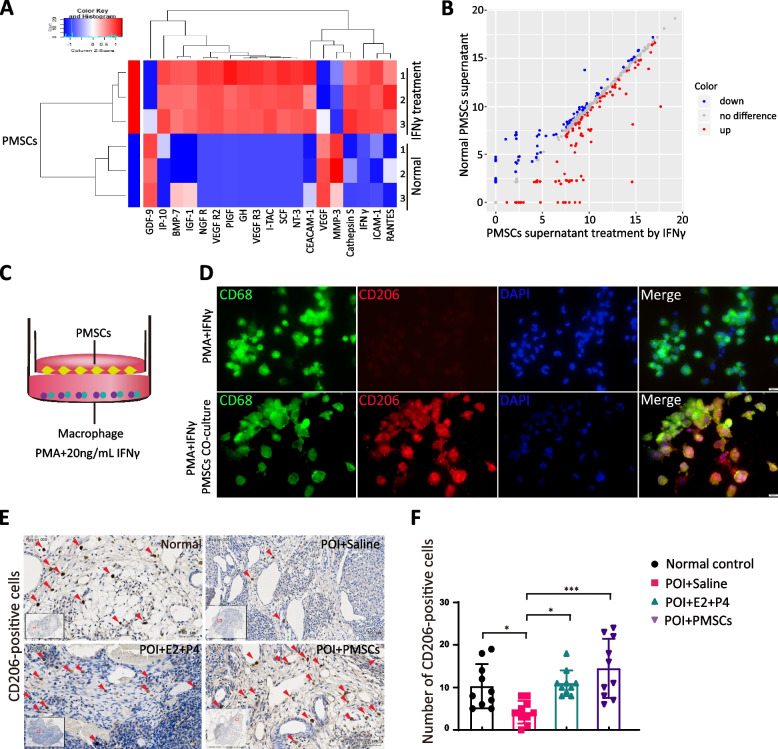
The number of M2 macrophages was increased by PMSCs in vitro and in vivo. **A** Cluster analysis by cytokine array showing the difference in paracrine factors of PMSCs after IFNγ-stimulation. **B** Volcano map. **C** Model of co-culture of macrophages and PMSCs. **D** Immunofluorescence analysis of macrophage phenotype, CD206, for M2 macrophages (*red*) and nuclear staining (*blue*). **E** The IHC staining of CD206 in each group rat ovary. Scale bar, 200 μm. **F** Quantification of macrophage phenotypes. The number of M2-like macrophages increased dramatically in the PMSCs group. Data are presented as the mean ± SD. *p < 0.05, **p < 0.01, ***p < 0.001, ns, not significant. Data are means of three independent experiments in each group

When PMSCs were cultured with IFN-γ for 48 h, the level of BMP-7, PIGF, IGF, SCF, cathepsin S and GH that promote macrophage proliferation and M2 polarization was significantly upregulated. It was observed that gene expression of adhesion molecules and macrophage chemotactic factors, such as CEACAM-1, ICAM-1, IP-10, I-TAC, RANTES and NT-3 was also enhanced (Fig. 5A, B). When activated macrophages were co-cultured with PMSCs, the differentiation of CD206^+^ macrophages was significantly increased (Fig. 5C, D). We also observed a higher number of CD206^+^ cells colonizing the ovaries of rats transplanted with PMSCs compared to the saline control group (*p* < 0.01) (Fig. 5E, F). Thus, PMSCs have a significant inhibitory effect on the M1 polarization of macrophages stimulated by IFNγ.

### PMSCs alleviate inflammasome-induced pyroptosis by down-regulating pro-inflammatory factors from macrophage

**Fig. 6:**
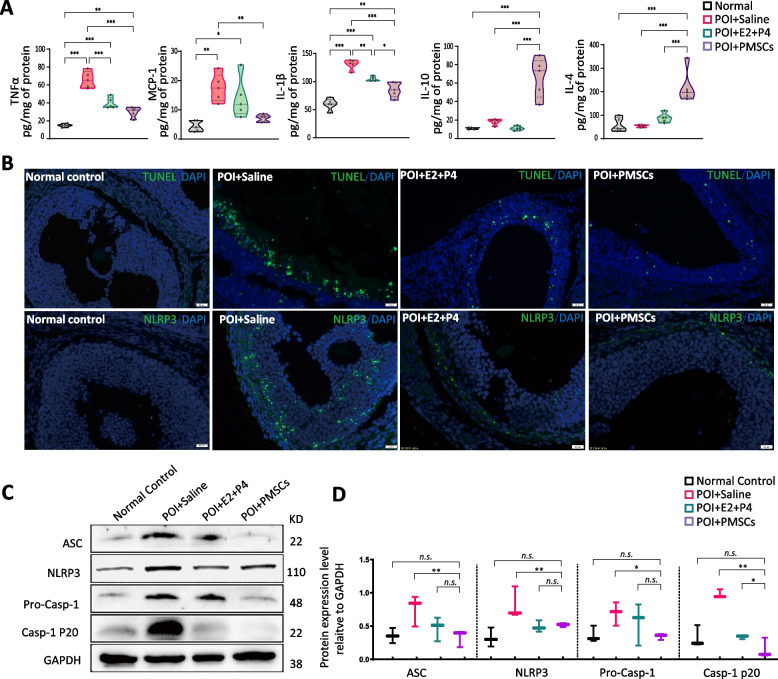
Expression of NLRP3 inflammasome pathway and related inflammatory factors. **A** Inflammation-related cytokine expression detected by ELISA. **B** TUNEL staining for apoptosis and immunofluorescence assay for NLRP3. Scale bar, 100 µm **C**, **D** Immunoblot analysis of NLRP3 inflammasome pathway and related inflammatory factors. Data are mean ± SD. *p < 0.05, **p < 0.01, ***p < 0.001, ns, no significance. Data are means of three independent experiments in each group

PMSC transplantation significantly downregulated the level of the inflammatory factors, IL-1β and TNF-α in ovarian tissues and MCP-1secretion in the POI model (*p* < 0.05). The level of IL-4 and IL-10 secretion was significantly upregulated by PMSC treatment (*p* < 0.05) (Fig. 6A). TUNEL-positive apoptotic cells were identified in POI model rats and the percentage of positive cells was increased compared with the PMSC and control groups. The results of immunofluorescence on ovarian sections showed an increase in NLRP3^+^ cells in the POI rats. The PMSC-transplant group had significantly lower NLRP3 inflammasome expression compared to the control and hormone groups. The extracted ovarian proteins were subjected to immunoblotting and quantitation (Fig. 6B, C). Compared to the normal group, the POI rat ovaries showed increased expression of NLRP3 and ASC. The expression of NLRP3 in the hormone group and the PMSC group decreased significantly. The PMSC treatment group also had lower expression of ASC and caspase-1 (both precursor and cleaved).

### PMSCs regulate the expression of the NLRP3 inflammasome by inhibited NFκB pathway activation

**Fig. 7:**
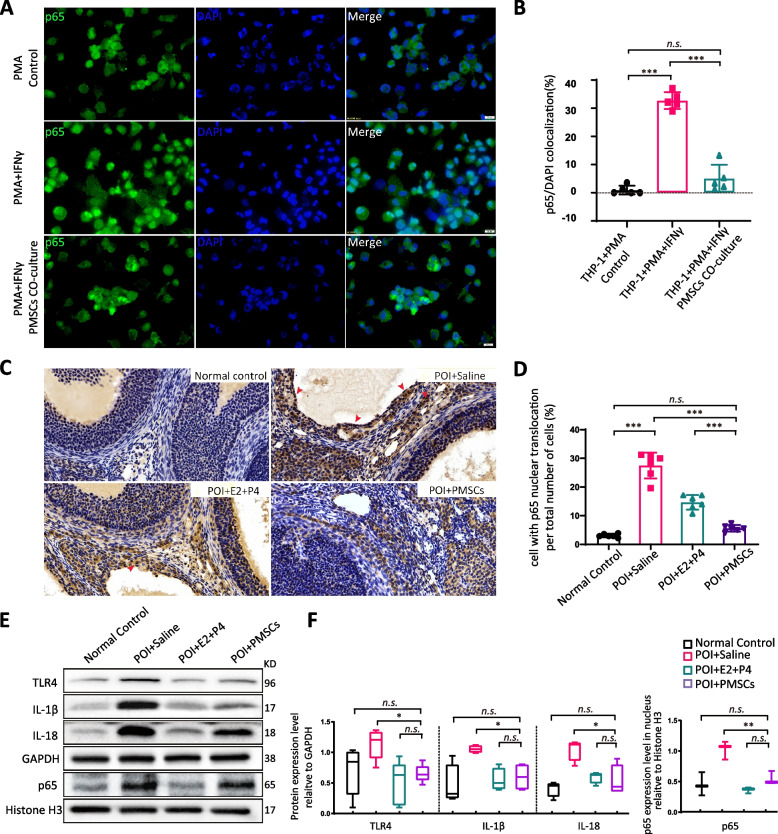
Activation of teNFκB pathway in vitro and in vivo. **A**, **B** Immunofluorescence assay for nuclear translocation of p65 in macrophages. Scale bar: 20 µm. **C**, **D** Immunohistochemistry assay for nuclear translocation of p65 in ovarian. Scale bar: 20 µm. **E**, **F** Immunoblot analysis of NFκB pathway and related inflammatory factors. Data are mean ± SD. *p < 0.05, **p < 0.01, ***p < 0.001, ns, no significance. Data are means of three independent experiments in each group

P65 nuclear staining was observed in macrophages after activation by IFN-γ but not in the control groups. The co-culture with hPMSC showed significantly reduced nuclear translocation of p65, indicating that NFκB pathway activity was significantly inhibited (Fig. 7A,B). The results of immunohistochemistry on ovary sections are shown in Fig. 7C. The staining for p65 in POI rats was primarily positive for the nuclear localization compared with the normal group. The PMSC transplant group had a significantly lower cell count for P65 nuclear staining compared to the control and hormone groups (Fig. 7D). Western blot indicated that VCD treatment increased the protein expression of TLR4/NFκB signaling pathway, such asTLR4, IL-1β, IL-18 and p65 in the POI saline group (*p* < 0.05), as well as, PMSC transplanted was decreased the expression of these proteins (Fig. 7E, F), indicating that NFκB pathway activity was significantly inhibited by immunomodulatory function of PMSC.

## Discussion

Countries around the world are making efforts to restore the birth population. However, in addition to facing the choice of “to give birth or not to give birth”, those women who “want to give birth but cannot” still encounter the difficulties of childbearing. From a clinical point of view, the ovarian aging process that results in impaired female fertility is currently pharmacologically uncontrollable and presents health threats to perimenopausal women such as osteoporosis and cardiovascular disease [15].

POI induced by 4-vinylcyclohexene diepoxide (VCD) has been increasingly used in recent years as a model for testing various therapies. In previous research, it was found that long-term, high-dose VCD not only kills small follicles as part of the pathogenesis of premature ovarian failure, but also accelerates the development and discharge of follicles in the growth phase [14]. Combined exposure to VCD and phthalates significantly reduced the numbers of primary follicles and consequently increased the risk of premature menopause; combined exposure to phthalates and VCD in early menopausal women is likely to aggravate POI [16]. We found that VCD had the advantages of safety, strong alignment, and good success rate in producing a POI model.

Estrogen replacement therapy (HRT) is very important for young patients with POI because it can relieve symptoms of low estrogen and can prevent long-term complications. But long-term HRT also has certain risks, such as the occurrence of endometrial cancer and breast cancer [1]. Mesenchymal stem cell transplantation is considered to be a very promising treatment strategy for reversing the negative effects of POI [4]. One of the key events that contribute to ovarian aging includes follicular atresia as it is associated with the pyroptosis and necrosis of granulosa cells and thecal cells in aging ovaries. Follicular atresia shows many pathophysiological alterations associated with physiological aging such as mitochondrial dysfunction, oxidative stress, and inflammation.

The anti-inflammatory activity of PMSCs has been demonstrated by modification of the type of paracrine inflammatory factors caused by proliferation and differentiation of lymphocytes and macrophages [17]. In this study, we focused on the paracrine effect of PMSCs on macrophage polarization in the IFN-γ-suffused ovarian microenvironment. IFN-γ plays a key role in activation of cellular immunity and the paracrine profile of PMSCs was markedly different after IFN-γ stimulation. BMP-7, placental growth factor (PIGF), IGF, SCF, and cathepsin S were up-regulated in PMSCs. BMP-7 significantly reduced pro-inflammatory M1 macrophages and increased anti-inflammatory M2 macrophages in BMP-7-treated mice [18]. Some studies indicated that PIGF [19], IGF1[20], SCF [21] and GH [22] reduced inflammation and played a critical role in polarizing the M1/M2 phenotypes via specific growth factor receptor pathways. Cathepsin S activity has been shown to be necessary for maintaining the TAM phenotype by profound metabolic changes in macrophages. More importantly, there were some adhesion molecules and macrophage chemotactic factors that were enhanced in stimulated PMSCs, such as CEACAM-1, ICAM-1, IP-10, I-TAC, RANTES and NT-3. These cytokine affected the migration and colonization of monocytes and macrophages at sites of injury.

The NLRP3 inflammasome recognizes a large and highly diverse set of agonists that respond to pathogen invasion, environmental stress, and tissue pathology. Studies have evaluated the role of the NLRP3 inflammasome in ovarian aging and female fertility[23, 24]. Age-dependent increased expression of NLRP3 in the ovary was observed in WT mice during reproductive aging [8]. As an important part of the inflammatory response, the inflammasome is a protein complex several microns in diameter that mainly includes receptor protein (receptor), adaptor protein (adaptor), ASC and the downstream caspase-1. After the receptor protein is activated by an agonist, it will attract ASC and caspase-1 to assemble into inflammasomes, thereby inducing self-cleavage and activation of caspase-1. Active caspase-1promotes the maturation and secretion of pro-inflammatory cytokines including IL-1β and IL-18, but it also triggers pyroptosis, which disposes of damaged cells and pathogens [25]. Multiple molecular or cellular events, including changes in ion flux, mitochondrial dysfunction, reactive oxygen species (ROS) generation, and lysosomal damage, have been shown to activate the NLRP3 inflammasome [6]. The active NLRP3 inflammasome causes follicular dysfunction and turns on the ovarian fibrosis signaling pathway [23]. There are currently few reports confirming that PMSCs protect ovaries against the damaging effects of POI, and it is unclear whether the mechanism involves suppression of the NLRP3 inflammasome by PMSCs. In this study, we clearly demonstrated that pyroptotic factors, such as NLRP3, ASC, caspase-1and IL-1β in the ovary, were also activated by VCD. Aged ovarian tissue is exposed to priming stimuli, such as ligands for toll-like receptors (TLRs), NLRs (e.g. NOD1 and NOD2), ROS or inflammatory cytokines, which activate the transcription factor NF-κB. NF-κB upregulates the expression of NLRP3 and promotes inflammasome formation and conversion of pro-IL-1β. Downstream maturation and release of IL-1and IL-18 continues to be involved in the feedback activation of the NLRP3 inflammasome via the TLR4/NF-κB pathway, further exacerbating inflammation. In our experiments, immunohistochemistry and western blot were used to quantitate NLRP3 inflammasome protein levels in the ovarian tissues of rats in each group. The expression of NLRP3, caspase-1, IL-1β, and IL-18 were measured to indirectly detect the level of inflammasome activity. At the same time, the nuclear translocation of NF-κB and the NF-κB signaling pathway activity were detected in ovarian cells. The results of that experiment showed that the expression levels of NLRP3, ASC, caspase-1, IL-1β and IL-18 decreased significantly after the injection of PMSCs into POI rats. There were a large number of NF-κB nuclear translocations in the ovarian tissue of the rats in the POI group, but the level of NF-κB was significantly reduced by PMSCs transplantation. These results suggest that the activity of the NF-κB inflammatory pathway was significantly decreased, which should inhibit activation of NLRP3 inflammasomes in damaged ovaries. The inhibition of inflammatory pathways may be caused by a reduction in the secretion of inflammatory factors in the ovarian microenvironment, and thus play a role in preventing reproductive harm from VCD-induced POI [26–28]. In contrast, PMSC transplantation restored the hormone secretion function of granulosa cells and theca cells by inhibiting the expression of pyroptosis proteins such as NLRP3, ASC, caspase-1and IL-1β.

Thus, the results of this experiment show that PMSCs can inhibit the activation of ovarian NLRP3 inflammasomes and decrease the degree of microenvironment inflammation and pyroptotic death of ovarian GCs in POI rats. During the development of POI, follicular dysfunction and anovulation are closely related to ovarian fibrosis. Numerous studies suggest that persistent inflammation contributes to ovarian injury. The findings of the present study indicate that activation of the NLRP3 inflammasome accelerates ovarian fibrosis in POI rats. Thus, the NLRP3 inflammasome is implicated as a potential target in the prevention of ovarian fibrosis progression. In this study, our findings revealed a novel mechanism by which VCD activated the NLRP3 inflammasome causing pro-inflammatory factor secretion, and driving follicular dysfunction and ovarian fibrosis; however, administering PMSCs significantly improved ovarian function by blocking this positive feedback loop.

## Conclusions

Our findings revealed a novel mechanism of follicular dysfunction and ovarian fibrosis via activation of the NLRP3 inflammasome followed by secretion of pro-inflammatory factors. Transplantation of PMSCs into POI rats suppressed pro-inflammatory factor production through enhanced macrophages M2 polarization, inhibited NLRP3 inflammasome formation and pyroptosis, and improved ovarian function. POI has become a disease that seriously endangers women’s reproduction and health. In the future, the mechanism of the pathogenesis of POI, the protection of ovarian function in groups at high risk of POI, and the development of new POI therapies should be the focus and direction of research to lay the foundation for the early diagnosis and mitigation of POI.

## Data Availability

All data and models generated or used during the study appear in the submitted article. The detailed datasets used or analysed during the current study are available from the corresponding author on reasonable request.

## Abbreviations

PMSCs: Placental mesenchymal stem cells
POI: Premature ovarian insufficiency
VCD: 4-Vinylcyclohexene diepoxide
FSH: Follicle stimulating hormone
GCs: Granulosa cells
E2: Estradiol
P4: Progesterone
AMH: Serum anti-müllerian hormone
17αOHP: 17-α-Hydoxy progesterone
LH: Luteinizing hormone
IL-1β: Interleukin-1β
NLRP3: NOD-like receptor thermal protein domain associated protein 3
HE: Hematoxylin and Eosin
SPF: Specific Pathogen Free
SD: Sprague–Dawley
MLC: Mixed-lymphocyte culture
PBMCs: Peripheral blood monocytes
IFN-γ: Interferon gamma
IL-2: Interleukin-2
IL-10: Interleukin-10
NF-κB: Nuclear Factor Kappa Beta
PFA: Paraformaldehyde
VG: Van Gieson
RIPA: Radio Immunoprecipitation Assay Lysis
TBST: Tris Buffered Saline with Tween® 20
PVDF: Polyvinylidene Fluoride
GAPDH: Glyceraldehyde-3-phosphate dehydrogenase
PBS: Phosphate-buffered saline
NC: Negative control
HRT: Estrogen replacement therapy
ROS: Reactive oxygen species
TLRs: Toll-like receptors
ASC: Apoptosis-associated speck-like protein containing CARD

## References

[1] Chon SJ, Umair Z, Yoon MS (2021). Premature Ovarian Insufficiency: Past, Present, and Future. Front Cell Dev Biol.

[2] Ulin M, Cetin E, Hobeika E, Chugh RM, Park HS, Esfandyari S, Al-Hendy A (2021). Human mesenchymal stem cell therapy and other novel treatment approaches for premature ovarian insufficiency. Reprod Sci.

[3] Shin EY, Kim DS, Lee MJ, Lee AR, Shim SH, Baek SW, Han DK, Lee DR (2021). Prevention of chemotherapy-induced premature ovarian insufficiency in mice by scaffold-based local delivery of human embryonic stem cell-derived mesenchymal progenitor cells. Stem Cell Res Ther.

[4] Polonio AM, Garcia-Velasco JA, Herraiz S (2020). Stem Cell Paracrine Signaling for Treatment of Premature Ovarian Insufficiency. Front Endocrinol (Lausanne).

[5] Sen Halicioglu B, Saadat K, Tuglu MI (2022). Adipose-derived mesenchymal stem cell transplantation in chemotherapy-induced premature ovarian insufficiency: the role of Connexin and Pannexin. Reprod Sci.

[6] Zhou R, Yazdi AS, Menu P, Tschopp J (2011). A role for mitochondria in NLRP3 inflammasome activation. Nature.

[7] Li Z, Zhang M, Tian Y, Li Q, Huang X (2021). Mesenchymal Stem Cells in Premature Ovarian Insufficiency: Mechanisms and Prospects. Front Cell Dev Biol.

[8] Navarro-Pando JM, Alcocer-Gomez E, Castejon-Vega B, Navarro-Villaran E, Condes-Hervas M, Mundi-Roldan M, Muntane J, Perez-Pulido AJ, Bullon P, Wang C (2021). Inhibition of the NLRP3 inflammasome prevents ovarian aging. Sci Adv.

[9] Zhu Y, Yang Y, Zhang Y, Hao G, Liu T, Wang L, Yang T, Wang Q, Zhang G, Wei J (2014). Placental mesenchymal stem cells of fetal and maternal origins demonstrate different therapeutic potentials. Stem Cell Res Ther.

[10] Shao F, Fitzgerald KA (2022). Molecular mechanisms and functions of pyroptosis. J Mol Biol.

[11] Jo EK, Kim JK, Shin DM, Sasakawa C (2016). Molecular mechanisms regulating NLRP3 inflammasome activation. Cell Mol Immunol.

[12] Yuan X, Li T, Shi L, Miao J, Guo Y, Chen Y (2021). Human umbilical cord mesenchymal stem cells deliver exogenous miR-26a-5p via exosomes to inhibit nucleus pulposus cell pyroptosis through METTL14/NLRP3. Mol Med.

[13] Na L, Wang S, Liu T, Zhang L (2020). Ultrashort Wave Combined with Human Umbilical Cord Mesenchymal Stem Cell (HUC-MSC) Transplantation Inhibits NLRP3 Inflammasome and Improves Spinal Cord Injury via MK2/TTP Signalling Pathway. Biomed Res Int.

[14] Cao LB, Leung CK, Law PW, Lv Y, Ng CH, Liu HB, Lu G, Ma JL, Chan WY (2020). Systemic changes in a mouse model of VCD-induced premature ovarian failure. Life Sci.

[15] Stevenson JC, Collins P, Hamoda H, Lambrinoudaki I, Maas A, Maclaran K, Panay N (2021). Cardiometabolic health in premature ovarian insufficiency. Climacteric.

[16] Tran DN, Jung EM, Yoo YM, Ahn C, Kang HY, Choi KC, Hyun SH, Dang VH, Pham TN, Jeung EB (2018). Depletion of follicles accelerated by combined exposure to phthalates and 4-vinylcyclohexene diepoxide, leading to premature ovarian failure in rats. Reprod Toxicol.

[17] Li K, Yan G, Huang H, Zheng M, Ma K, Cui X, Lu D, Zheng L, Zhu B, Cheng J (2022). Anti-inflammatory and immunomodulatory effects of the extracellular vesicles derived from human umbilical cord mesenchymal stem cells on osteoarthritis via M2 macrophages. J Nanobiotechnology.

[18] Shoulders H, Garner KH, Singla DK (2019). Macrophage depletion by clodronate attenuates bone morphogenetic protein-7 induced M2 macrophage differentiation and improved systolic blood velocity in atherosclerosis. Transl Res.

[19] Sunakawa Y, Stintzing S, Cao S, Heinemann V, Cremolini C, Falcone A, Yang D, Zhang W, Ning Y, Stremitzer S (2015). Variations in genes regulating tumor-associated macrophages (TAMs) to predict outcomes of bevacizumab-based treatment in patients with metastatic colorectal cancer: results from TRIBE and FIRE3 trials. Ann Oncol.

[20] Ji Y, Duan W, Liu Y, Liu Y, Liu C, Li Y, Wen D, Li Z, Li C (2018). IGF1 affects macrophage invasion and activation and TNF-alpha production in the sciatic nerves of female SOD1G93A mice. Neurosci Lett.

[21] Xie M, Zhang S, Dong F, Zhang Q, Wang J, Wang C, Zhu C, Zhang S, Luo B, Wu P (2021). Granulocyte colony-stimulating factor directly acts on mouse lymphoid-biased but not myeloid-biased hematopoietic stem cells. Haematologica.

[22] Spadaro O, Goldberg EL, Camell CD, Youm YH, Kopchick JJ, Nguyen KY, Bartke A, Sun LY, Dixit VD (2016). Growth Hormone Receptor Deficiency Protects against Age-Related NLRP3 Inflammasome Activation and Immune Senescence. Cell Rep.

[23] Wang D, Weng Y, Zhang Y, Wang R, Wang T, Zhou J, Shen S, Wang H, Wang Y (2020). Exposure to hyperandrogen drives ovarian dysfunction and fibrosis by activating the NLRP3 inflammasome in mice. Sci Total Environ.

[24] Lliberos C, Liew SH, Mansell A, Hutt KJ (2020). The inflammasome contributes to depletion of the ovarian reserve during aging in Mice. Front Cell Dev Biol.

[25] Tschopp J, Schroder K (2010). NLRP3 inflammasome activation: the convergence of multiple signalling pathways on ROS production?. Nat Rev Immunol.

[26] Cruz-Barrera M, Florez-Zapata N, Lemus-Diaz N, Medina C, Galindo CC, Gonzalez-Acero LX, Correa L, Camacho B, Gruber J, Salguero G (2020). Integrated analysis of transcriptome and Secretome from umbilical cord mesenchymal stromal cells reveal new mechanisms for the modulation of inflammation and immune activation. Front Immunol.

[27] Liu C, Xu Y, Lu Y, Du P, Li X, Wang C, Guo P, Diao L, Lu G (2022). Mesenchymal stromal cells pretreated with proinflammatory cytokines enhance skin wound healing via IL-6-dependent M2 polarization. Stem Cell Res Ther.

[28] Lv H, Yuan X, Zhang J, Lu T, Yao J, Zheng J, Cai J, Xiao J, Chen H, Xie S (2021). Heat shock preconditioning mesenchymal stem cells attenuate acute lung injury via reducing NLRP3 inflammasome activation in macrophages. Stem Cell Res Ther.

